# Congenital Microphthalmia with Orbital Cyst: A Case Series

**DOI:** 10.31729/jnma.4473

**Published:** 2019-06-30

**Authors:** Diwa Hamal, Prerna Arjyal Kafle, Priza Poudyal, Rohit Saiju, KC Hony, Santosh Kafle

**Affiliations:** 1 Department of Oculoplasty, Biratnagar Eye Hospital, Biratnagar Nepal; 2 Department of Laboratory Medicine, Biratnagar Eye Hospital, Biratnagar Nepal; 3 Department of Oculoplasty, Tilganga Institute of Ophthalmology, Gaushala, Kathmandu, Nepal

**Keywords:** *congenital anomaly*, *cyst*, *eye*, *microphthalmos*

## Abstract

Microphthalmos results from incomplete invagination of the optic vesicle or closure of the embryonic fissure. We present three patients with unilateral congenital microphthalmia with cyst. None of them had vision in the affected eye since birth. There was gradually increasing left eye orbital mass encroaching towards lower fornix and lower eyelid ectropion. On examination and investigations, patients had large orbital cyst with microphthalmia pushing the eyeball superiorly and posteriorly in affected orbit. Microphthalmic globe with cyst was surgically excised and histopathologically studied. Orbital cavity was big enough to occupy the conformer and the prosthetic eye after 6 weeks. Diagnosis was confirmed as large communicating orbital cyst with microphthalmia without systemic association in all the patients. None of the mothers of patients had regular antenatal check up. All the parents had consanguineous marriage. Antenatal check up with ultrasound at 14 to16 weeks of pregnancy is important for genetic counselling. Targeted abdominal ultrasound examination of pregnant women focused on the orbital region of foetus is recommended, in mothers who have children with congenital eye anomalies.

## INTRODUCTION

Microphthalmia with cyst is a rare condition. About 150 cases have been reported and only one-third of them were bilateral. It may be genetic or non-genetic, unilateral or bilateral, and isolated or as a component of syndrome. Extra-ocular malformations are more frequently associated with bilateral cases.^1^ Orbital cyst with microphthalmos occurs due to defective closure of embryonic fissure at 7-14mm stage.^2,3^ Prevalence of congenital microphthalmos is 1.4-3.5 per 10000 births.^4^

We present three patients, two male and one female within age range of 3-24 years diagnosed as unilateral congenital microphthalmia with cyst involving right eye (RE) in one patient and left eye (LE) in two that was surgically removed and histopathologically studied.

## CASE REPORT

### CASE 1

A 3 years old male presented with gradually increasing mass in LE since birth. On examination of his LE, the mass was extending to lower fornix but detailed anatomy could not be distinguished. There was no perception of light (NPL) in his LE. Mass was soft, cystic under cover of conjunctiva, trans- illumination test was positive. His palpebral fissure could not be opened fully. Palpebral fissure height was 6mm in LE. There was absence of both upper and lower fornix. Eyelashes were present normally in both upper and lower eyelids in LE. There was mild ectropion of left lower lid. His vision in right eye was 6/6. Both anterior and posterior segments were normal in right eye (RE). There was history of consanguineous marriage in parents. Age of mother was 26. The child was delivered by normal vaginal delivery with irregular ANC visit. The patient's parents do not give history of abdominal ultrasound during antenatal period.

Contrast enhanced computed tomography (CECT) scan revealed large well defined hypodense cystic lesion measuring 2.7x2.1cm in left orbit causing compression and displacement of orbital nerve, bony orbit laterally leading to atrophic left globe postero superiorly located with calcification. There was no CNS involvement. Total excision of the cyst with communicating microphthalmic globe was done and sent for histopathological examination (HPE). The large orbital cyst in this patient has played an import^^rae in socket expansion thus the socket was big enough to accommodate a conformer.

**Figure 1.**
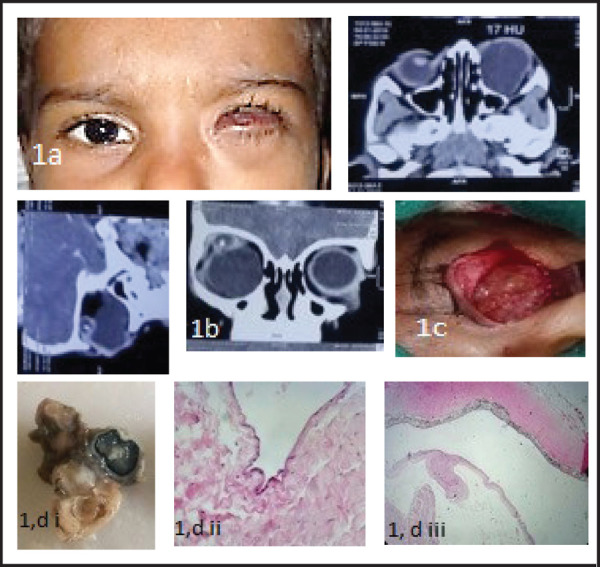
(a) Clinical photograph showing left congenital microphthalmia with orbital cyst. (b) Computer tomography (CT) orbit with a large cystic lesion occupying the left inferior orbit displacing the microphthalmic globe superiorly. (c) Inferiorly located large multiloculated cyst in the left orbit. The globe was totally covered by the cyst. (d) Gross: i. Cut surface shows an eyeball callot with attached cyst. Microscopy: ii. Shows cystic lining of tissue with oedematous fibrocollagenous stroma (H&E stainx40). iii. Shows layers of sclera, choroid and retinal layers (H&E stain x10). Impression: suggestive of Micropthalmia with cyst.

The gross and HPE showed an eye ball with deformed looking structure measuring 2.9x3x1.2cm with corneal tissue 0.7mm in diameter, lined by stratified squamous epithelium, the sub-epithelial stroma reveal scattered few congested vascular channels along with some mixed chronic inflammatory cells. There was presence of degenerate layer of retina, iris (focally) and acellular fibro collagenous choroid-like layers. Sections from cystic structure attached with the small eyeball shows acellular fibro collagenous tissue mimicking the oedematous choroidal layer along with presence of focal mixed chronic inflammatory cells infiltrate. That confirmed it to be microphthalmia with communicating cyst.

### CASE 2

A 17 years old female had similar presentations as the first patient with mass in LE since birth. On examination of her LE her anatomy could not be distinguished. There was no perception of light in her LE. Mass was soft, cystic under cover of conjunctiva, trans-illumination test was positive. Her palpebral fissure could not be opened fully. Palpebral fissure height was 4mm in left eye. There was absence of both upper and lower fornix. Eyelashes were present normally in both upper and lower eyelids in LE. There was ectropion of left lower eyelid. Her vision in right eye was 6/6. Both anterior and posterior segments were normal in RE. There was history of consanguineous marriage in parents.

CECT scan revealed small left globe with a focal calcification at its optic disc, thin optic nerve, extra ocular muscles and 36x31x27 mm non-enhancing extra- conal hypodense cystic mass (similar in attenuation to the vitreous chamber in infero-lateral quadrant of left orbit, displacing the globe superomedially causing widening of the bony orbit. Suggesting left microphthalmia with cystic.

Total excision of the cyst with microphthalmic globe was done and sent for HPE. The orbital cyst in this patient has played an important role in socket expansion thus the socket was big enough to accommodate a conformer. The HPE showed it to be consistent with microphthalmia with cyst.

**Figure 2.**
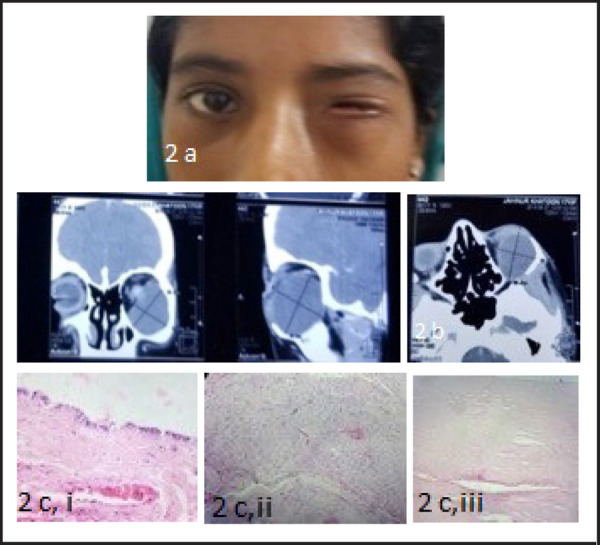
(a) Clinical photograph showing left congenital microphthalmia with orbital cyst. (b) CT orbit of the second patient with a large cystic lesion occupying the left inferior orbit displacing the microphthalmic globe superiorly. (c) Gross: Two fragmented soft tissue were received. Microscopy: i. Shows cystic lining of tissue with oedematous fibro collagenous wall (H&E x40) ii. Shows corneal oedematous stroma (H&E stain x40) iii. Shows neural component mimicking retinal layers (H&E stain x40). Impression: Benign cystic lesion, most consistent with Micropthalmia with cyst.

### CASE 3

A 25 years old male had similar presentations with mass in RE since birth. On examination of his right eye intra ocular anatomy could not be examined. There was no perception of light in his RE. Mass was soft, cystic under cover of eye lid, trans-illumination test was positive. His right eye palpebral fissure could not be opened. There was absence of both upper and lower fornix in his right eye. Eyelashes were present sparsly in both lower eye lid in RE.

The lower lid completely covered the upper lid in RE. His vision in LE was 6/6. Both anterior and posterior segments were normal in LE. There was history of consanguineous marriage in his parents.

Computed tomography scan revealed 35x34x29mm soft mass intraconally in right orbital cavity displacing the atrophic globe superiorly with a focal calcification of 5x 5 mm with thining of right optic nerve. Extra ocular muscles were visualized except inferior rectus was seen inseperable from the mass. There was no destruction or erosion of right bony orbit. Suggesting right microphthalmia with cystic orbital lesion.

Total excision of the cyst with microphthalmic globe was done and sent for histopathological examination (HPE). The HPE showed it to be consistent with microphthalmia with cyst.

All three patients were advised with oral and systemic antibiotic, NSAID. Post operatively patient was advised for ocular prosthesis.

**Figure 3.**
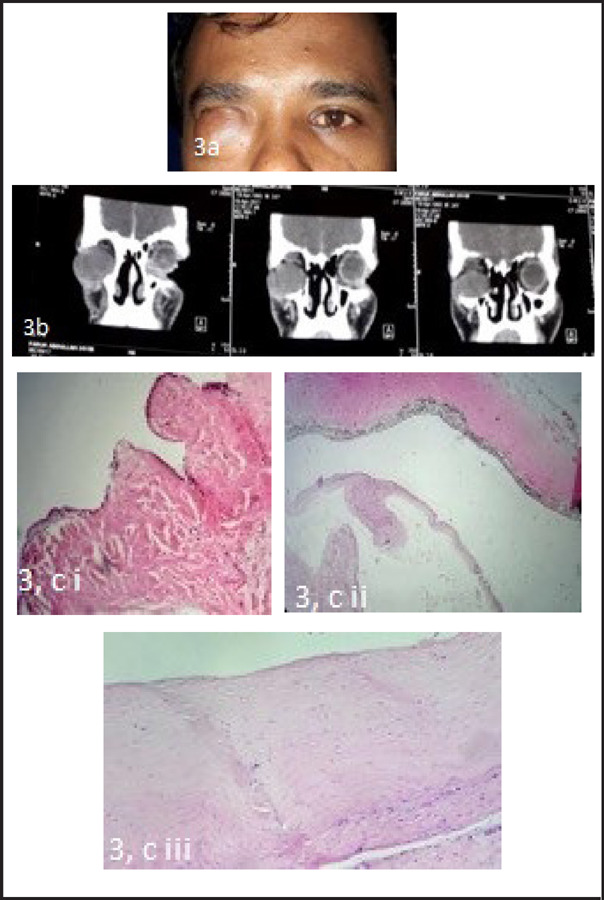
(a) Clinical photograph showing right congenital microphthalmia with orbital cyst. (b) Showing CT orbit with a large cystic lesion occupying the right inferior orbit displacing the microphthalmic globe superiorly. (c) Gross: Two fragmented soft tissue were received. Microscopy: i. Shows cystic lining of tissue with oedematous fibrocollagenous wall (H&E x40). ii. Shows edematous corneal stroma. iii. Shows the neural component mimicking retinal layers (H&E stain x10). Impression: Benign cystic lesion, most consistent with micropthalmia with cyst.

## DISCUSSION

Patients with microphthalmia presents with spectrum of visual impairment from mild impairment to complete blindness. Patients can suffer from intellectual disability, if the anomaly presents as part of a syndrome. Prognosis is varied and patients usually require the support of a multidisciplinary team to manage the vision, for plastic surgery, intellectual and psychological aspects.^5^

The differential diagnosis for cystic anomalies include microphthalmia with cyst, congenital cystic eye, microphthalmia with cystic teratoma, ectopic brain tissue and meningo-encephalocele. Microphthalmos with cyst is distinguished on the basis of a small globe with a communicating channel between it and cyst.^6^ Sometimes, the congenital colobomatous microphthalmos with orbital cyst may not communicate with the eye or the central nervous system. Microphthalmia with cyst is non-hereditary, but has been described in siblings and with syndromes. Genetic disorders responsible for microphthalmos with cyst include autosomal recessive, autosomal dominant, and X linked syndromes. Once the condition has been identified, genetic counselling has to be given to the family.

Two unilateral cases (Jensen 1965; Fledelius 1996) and one bilateral case (Ehlers 1966) have previously been published.^7–9^ The previously presented cases of bilateral microphthalmia with cyst, the cysts had made contact with the eyes. Removal of both eye with cyst is the most frequent therapy.^10^ Congenital microphthalmos with cyst is defined in three categories, a relatively normal eye with a small cyst, clinically not apparent, an obvious cyst associated with a grossly deformed eye, lastly a large cyst which has pushed the globe backwards so that it is not visible clinically.^11^ Severe microphthalmia according to the anatomic appearance and severity of the reduction of the globe refers to a globe with a corneal diameter less than 4 mm and a total axial length less than 10 mm at birth.^12^

Presentation symptoms are generally congenital mass with increasing lower lid protrusion.^2,3^ Congenital micropthalmos with cyst can be isolated or associated with other ocular or systemic abnormalities. Congenital heart defects, central nervous system diseases, cleft lip/palate, pulmonary aplasia and renal agenesis was reported to be associated.^1,2,3,12,13,14^ Although systemic abnormalities associated with microphthalmos were not common in cases with bilateral cysts compared to cases with unilateral cysts in some reports.^4^ Regular antenatal checkup along with ultrasound is recommended in every pregnant women. Mashiach et al. described how transvaginal sonography at 14-16 weeks was helpful in detecting the eye abnormality in five cases in which at least one previous child in the family had the same congenital anomaly of eye.^15^ Searle A et al, advised to use a curvilinear transducer (2-9 MHz) and imaging orbits in both axial and coronal planes.^16^ This can be aided by using a trans vaginal probe in selected cases if it is difficult to visualize structures by the trans abdominal method. Three-dimensional reverse-face imaging should be obtained in suspected cases of anophthalmia or microphthalmia.

In all our cases, the cyst was grade 3 according to Elder classification as the large cyst had pushed the globe backwards so that it was not visible clinically. Excision of cyst along with the microphthalmic eye was done.

Management is not standard for every case and principally depends on visual potential, cyst size and orbital volume. Simple small cysts with normal ranged orbital volume should be observed while others treated surgically^2^. Surgery is usually recommended before school age for cosmetic appearance, since 90% orbital bone maturation was already completed until school age.^4,17^

Excision of the cyst alone may be performed if there is no contact with the eye or intracranially. Cystectomy has been performed in a limited number of cases.^18^ However, sparing the micropthalmic eye implies repeated follow-up consultations because cysts may recur and neoplasia may develop.^19^

## Consent

**JNMA Case Report Consent Form** was signed by the patient and the original is attached with the patient's chart.

## Conflict of Interest

**None.**
